# Creating a driving profile for older adults using GPS devices and naturalistic driving methodology

**DOI:** 10.12688/f1000research.9608.2

**Published:** 2016-12-07

**Authors:** Ganesh M. Babulal, Cindy M. Traub, Mollie Webb, Sarah H. Stout, Aaron Addison, David B. Carr, Brian R. Ott, John C. Morris, Catherine M. Roe

**Affiliations:** 1 Charles F. and Joanne Knight Alzheimer’s Disease Research Center, Department of Neurology, Washington University School of Medicine, St. Louis, MO, 63130, USA; 2 Data and GIS Services, University Libraries, Washington University in St. Louis, St. Louis, MO, 63130, USA; 3 Pathology and Immunology, Washington University School of Medicine, St. Louis, MO, 63110, USA; 4 Physical Therapy, Washington University School of Medicine, St. Louis, MO, 63110, USA; 5 Occupational Therapy, Washington University School of Medicine, St. Louis, MO, 63110, USA; 6 Departments of Medicine and Neurology, Divisions of Geriatrics and Nutritional Science/Neurorehabilitation, St. Louis, MO, 63110, USA; 7 Department of Neurology, Warren Alpert Medical School, Brown University, and Rhode Island Hospital, Providence, Rhode Island, USA

**Keywords:** naturalistic driving, global positioning data acquisition systems, geographic information system, in-vehicle technology, older adults, Alzheimer’s disease

## Abstract

**Background/Objectives**: Road tests and driving simulators are most commonly used in research studies and clinical evaluations of older drivers. Our objective was to describe the process and associated challenges in adapting an existing, commercial, off-the-shelf (COTS), in-vehicle device for naturalistic, longitudinal research to better understand daily driving behavior in older drivers.

**Design**: The Azuga G2 Tracking Device ^TM ^was installed in each participant’s vehicle, and we collected data over 5 months (speed, latitude/longitude) every 30-seconds when the vehicle was driven.

**Setting**: The Knight Alzheimer’s Disease Research Center at Washington University School of Medicine.

**Participants**: Five individuals enrolled in a larger, longitudinal study assessing preclinical Alzheimer disease and driving performance.  Participants were aged 65+ years and had normal cognition.

**Measurements**:  Spatial components included Primary Location(s), Driving Areas, Mean Centers and Unique Destinations.  Temporal components included number of trips taken during different times of the day.  Behavioral components included number of hard braking, speeding and sudden acceleration events.

**Methods**:  Individual 30-second observations, each comprising one breadcrumb, and trip-level data were collected and analyzed in R and ArcGIS.

**Results**: Primary locations were confirmed to be 100% accurate when compared to known addresses.  Based on the locations of the breadcrumbs, we were able to successfully identify frequently visited locations and general travel patterns.  Based on the reported time from the breadcrumbs, we could assess number of trips driven in daylight vs. night.  Data on additional events while driving allowed us to compute the number of adverse driving alerts over the course of the 5-month period.

**Conclusions**: Compared to cameras and highly instrumented vehicle in other naturalistic studies, the compact COTS device was quickly installed and transmitted high volumes of data. Driving Profiles for older adults can be created and compared month-to-month or year-to-year, allowing researchers to identify changes in driving patterns that are unavailable in controlled conditions.

## Background

The high risk of crashes and decline in driving ability among older adults with Alzheimer disease is well documented; the risk of injury and mortality from motor vehicle crashes increases with age and dementia severity ^1^. Our research program seeks to understand driving behavior among older adults, particularly as it occurs on a day-to-day basis as people travel in their own environments. However, evaluation of driving behavior in older adults largely occurs with methodologies that use controlled conditions such as on-the-road tests and driving simulators, and to a lesser extent, self-report and diaries ^2– 5^. To better meet our research needs, we explored newer methodologies to study naturalistic driving behavior longitudinally, in a cost effective and unobtrusive manner ^6^.

Recent technological advances in global positioning systems (GPS) and geographic information systems (GIS) techniques allow evaluation of driving behavior in the actual environments in which individuals drive ^7^. Newer in-vehicle GPS devices are unobtrusive and typically provide data on date, time, speed, longitude and latitude regarding where a vehicle is driven ^8, 9^. In-vehicle GPS/GIS devices are an emerging methodology employed to better understand driving *in situ* and compare differences between driver self-report and GPS data obtained from a vehicle ^10^. As a result, naturalistic driving research employing this methodology seeks to understand driving behavior by analyzing continuous, objective data collected by in-vehicle devices to determine patterns and the influence of personal, temporal and environmental factors ^8, 11^.

The evolving field of naturalistic driving and the proliferation of custom and commercial off the shelf (COTS) in-vehicle devices have resulted in numerous different outcomes and GIS analytical techniques ^9, 12^. However, some challenges accompany GPS data use, including extensive post-processing of large volumes of data, variability with temporal and spatial aspects of the data, and higher cost associated with the technology and data collection. Consequently, the monitoring periods in some recent studies using GPS and GIS are limited to capturing data for analysis from a timespan ranging from weeks to 2 months ^10, 12^. However these short periods may be too brief to capture relevant driving behaviors.

To more accurately monitor key driving naturalistic driving behaviors, we piloted a new methodology adapting a COTS in-vehicle device to study naturalistic driving behavior longitudinally, in a cost effective and unobtrusive manner. Our objective for this pilot is to describe methodological challenges associated with adapting a COTS in-vehicle device that captures and synthesizes GPS data for processing and analysis using GIS techniques. We also quantify spatial and temporal patterns associated with driving behavior to construct driver profiles to evaluate how driving behavior changes longitudinally.

## Methods

**Participant data.** Data were collected from participants enrolled in a longitudinal study assessing preclinical Alzheimer’s disease and driving performance (R01 AG043434) at Washington University School of Medicine in St. Louis. Participants had normal cognition, were 65 years or older, had a valid driver’s license, drove at least once per week in a non-adapted vehicle, met minimal visual acuity for state requirements, and had Alzheimer’s disease biomarkers (cerebrospinal fluid or brain imaging) objectively measured and available within the last two years. All study protocols, consent documents and questionnaires were approved by Washington University Human Research Protection Office.

**Data collection and processing.** We used the COTS Azuga G2 Tracking Device ^TM^ (Model 850: Azuga Inc, San Jose, California), which we refer to as a global positioning data acquisition system (GPDAS). The GPDAS plugs into the on-board diagnostic systems port (OBDII) and is powered by the vehicle’s battery. Installation requirements limit vehicles to those manufactured in 1996 or later since earlier years were not equipped with an OBDII port. Data (vehicle speed, latitude, longitude) were collected from the moment ignition was turned on and until it was turned off, with a collection interval set at every 30 seconds. Individual 30-second observations are referred to as a “breadcrumb”. Location data were also collected every three hours when the ignition was off. Additionally, aggressive driving incidents such as hard braking, speeding and sudden acceleration were recorded in the trip log. Data were collected and simultaneously transmitted via Bluetooth Low Energy to secured servers. On a daily basis, the data were aggregated by Azuga and made available for download via secured servers.

Two distinct file types available from Azuga were used in our analysis – Breadcrumb files and Activity files. Within the daily Breadcrumb comma separated values (csv) file, each row consisted of one observation ("breadcrumb"), typically at a 30 second interval for a specific vehicle at an instant of time. Each breadcrumb identified the vehicle by a 10-digit code and additionally reports latitude, longitude, vehicle speed, nearest address (reverse geocoded by Azuga), coordinated universal time (UTC) and date, odometer reading, and event type. The event type field identified whether the given breadcrumb was associated with a regular observation or special event such as ignition on/off or aggressive driving. The event type field could also contain codes indicating specific issues such as a low battery level in the vehicle, a connection or disconnection of the device, or a malfunction in the device hardware. Additional fields gave data about the peak speed and average speed of an over-speeding event, as well as initial and final speeds of braking or acceleration events characterized by a rapid change in the vehicle’s velocity.

The second file type received from Azuga was the daily Activity csv file. Each row in the Activity file represented one trip taken by a single vehicle. Available observations about each trip included the date and start time (in UTC), the starting and ending locations (latitude, longitude, and reverse-geocoded address), the duration/length of the trip in seconds and in distance (rounded to the nearest tenth of a kilometer, then reported in miles), the average and maximum vehicle speed, and the number/duration of aggressive driving events such as sudden acceleration, hard braking, and over-speeding. Preliminary data processing used a Powershell script to compare headers from the incoming Breadcrumb and Activity files to ensure the structure was consistent, and then combined the daily files from the time period of interest into two large comprehensive csv files (one each for Breadcrumbs and for trip-level Activity). These two large csv files were read into the statistical analysis program R as data tables for further analyses. For the remainder of the manuscript, the term *breadcrumb* refers to a single observation of one vehicle at a specific location and single moment in time, while a *trip* ****represents a set of locations (breadcrumbs) occurring between the ignition on and ignition off of a specific vehicle. Over the first five months, over 400,000 breadcrumbs representing approximately 12,000 trips were collected for the 20 vehicles.

**Figure 1.**
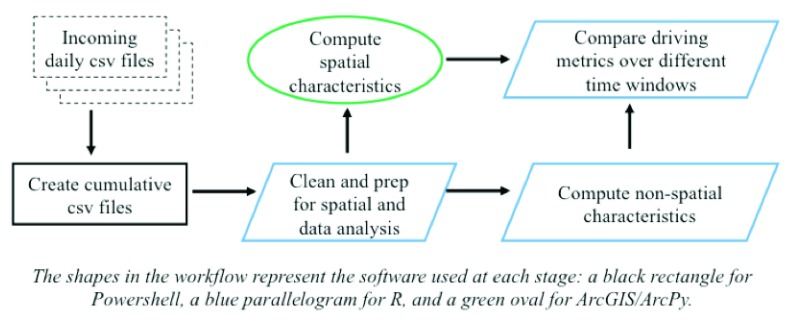
Data workflow required to generate driving metrics over different time scales.

Initial processing steps taken in R examined the condition of the incoming data for errors and anomalies, then created additional fields for use in aggregating the data, as well as the spatial processing stages. Since all times were reported in UTC and our participants were in the continental United States, time zone calculations were performed to accurately transform the incoming timestamp to local time. Many points in the Central Standard Time Zone were classified as such within R using a bounding rectangle with maximum/minimum latitude and longitude encapsulating the majority of the Central Standard Time Zone. For points close to the boundary of time zones, GIS was used to determine the appropriate zone. This was done by comparing the breadcrumb location against a set of polygons representing the extent of each time zone to determine in which time zone polygon the breadcrumb location fell in. Local time was needed to understand driving activity or avoidance during specific times of day (rush hour, daylight, etc.). The R package lubridate was used to convert UTC time to local time, while the R package RAtmosphere allowed for computations of sunrise and sunset at a given latitude/longitude. These computations were added as additional columns in the data tables. A summary of the workflow is given in Figure 1.

To clean the incoming data and prepare these for spatial processing, data were checked to ensure that two criteria were met: (1) each observation occurred within the continental United States and (2) no two observations for the same vehicle had identical timestamps. Certain device actions (being connected, disconnected, or plugged into a different vehicle) caused the GPDAS to report latitude and longitude values of 0, or in one case, those of a location in Egypt. Additionally, for some vehicles that started a trip immediately after plugging in the GPDAS, the time delay required to connect to a sufficient number of GPS satellites to register locational data caused a sequence of observations with latitude and longitude equal to 0. Due to uncertainty about the location of the vehicle at times where the latitude or longitude was reported outside the continental United States, those trips, including associated breadcrumbs were removed from analyses. The number of breadcrumbs impacted was less than 1.6% of all incoming breadcrumbs, with the vast majority of Figure 1 (6392 out of 6529) representing one vehicle whose GPDAS had a malfunction causing no locational data to be collected for multiple weeks. Removing the vehicle with a faulty GPDAS from the computation reduced the number of breadcrumbs removed by the first criteria to 137, less than 0.04% of the total number of breadcrumbs collected. The second criteria removed 12 breadcrumbs that were exact duplicates of other breadcrumbs.

Further data cleaning was required to compile a set of complete trips taken by each driver. Trip-level data were accessible in two ways from the incoming data stream. The Activity files contained summary information about the start, end, and length of each trip, while the Breadcrumb files offered a finer level of locational detail within the trip. Approximately 1.6% (n=203) of the incoming activity records contained NA values as latitude and longitude of the trip end. Typically this was caused by either a loss of GPS signal (such as parking in an underground structure) or a peculiarity of the incoming activity data stream, in which a second recorded trip start occurred several seconds after the first, which was then "abandoned" as a meaningful trip in the data stream. An additional 1.8% of reported trips (n=229) contained a value of 0 for the starting latitude or longitude. Most of these (217) were from the aforementioned known defective device that transmitted large numbers of zeros within the breadcrumb data. These were marked for removal.

**Analysis.** Data analysis and management for spatial operations in GIS, used ArcGIS 10.3.1 and the ArcPy Python site package (Environmental Systems Research Institute, Redlands, CA, USA). Spatial data were stored as feature classes in file geodatabase format. Time zone computations were exported from ArcGIS as a csv file and merged back in with the data table in R for further computations.

**Spatial analysis.** Using the latitude and longitude for each breadcrumb, point feature classes were created for each driver by exporting the results of the Make XY Event geoprocessing operation. These point feature classes served as the basis for all subsequent spatial analysis.

**Figure 2.**
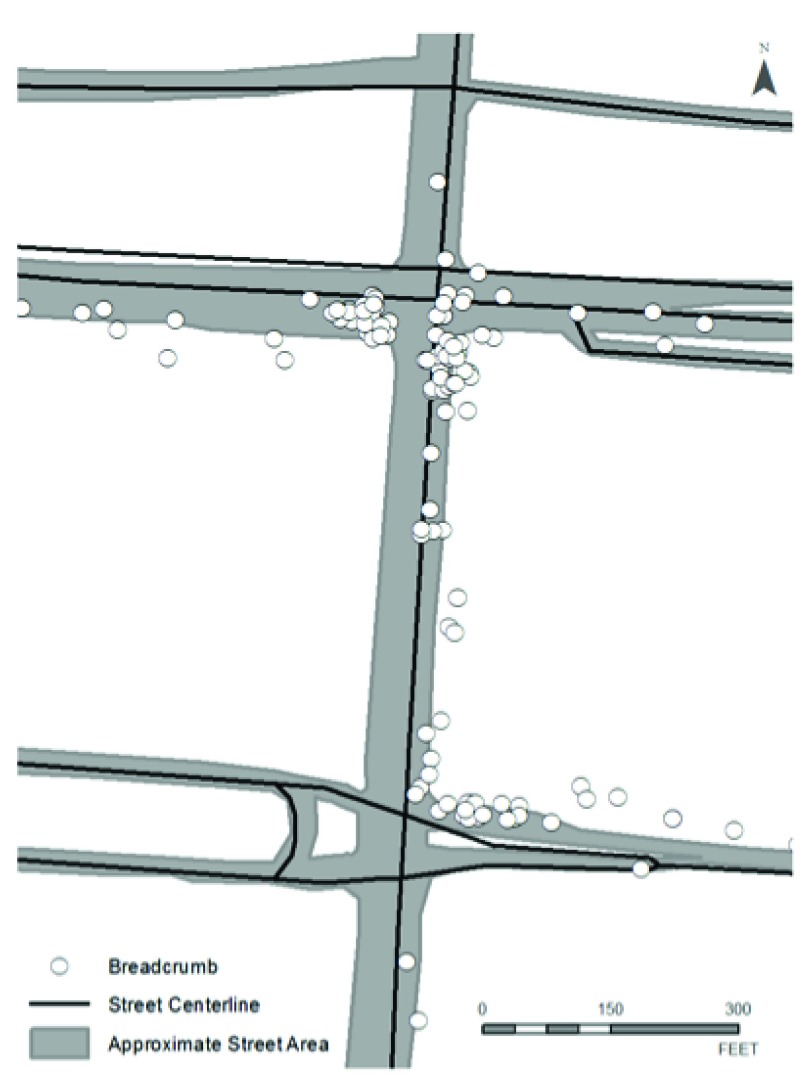
Breadcrumbs and street centerline features used in near analysis.

**Road analysis.** To determine the characteristics of the road over which the participant was traveling at the time of breadcrumb recording, proximity analysis was performed on each breadcrumb relative to a street centerline dataset. The Near geoprocessing operation was used to identify the street centerline feature closest to the street feature for each breadcrumb. The output of the Near geoprocessing operation is the addition of two attributes to the breadcrumb feature class. These attributes are NEARFID, the unique identifier of the nearest street feature, and NEARDIST, the distance from the target breadcrumb to the nearest street feature. The NEARFID value was used to retrieve attributes of the street feature nearest to the breadcrumb, such as the road name, Census Feature Class Code (CFCC), road type, and average speed (proxy for speed limit). Figure 2 shows a sample of breadcrumbs and their proximity to the street centerline features. Attributed values from the nearest street feature were applied to each breadcrumb using a series of Cursors. Cursors are iterator tools available in the ArcPy code library that can read, update and create features in existing spatial datasets (ArcGIS Help 2015).

**Driving Areas.** Driving Area was defined as the smallest polygon that encompassed all breadcrumbs for a driver during a given time period. The Minimum Bounding Geometry geoprocessing operation was used to produce convex hull polygons representing the weekly and monthly Driving Areas for each driver.

**Mean Center.** The Mean Center was defined as the geographic center of all breadcrumbs for a driver during a given time period. The Mean Center geoprocessing operation was used to produce points representing the weekly and monthly Mean Center for each driver. The operation was based on spatial location of the breadcrumbs only and was not weighted by any attribute.

**Figure 3.**
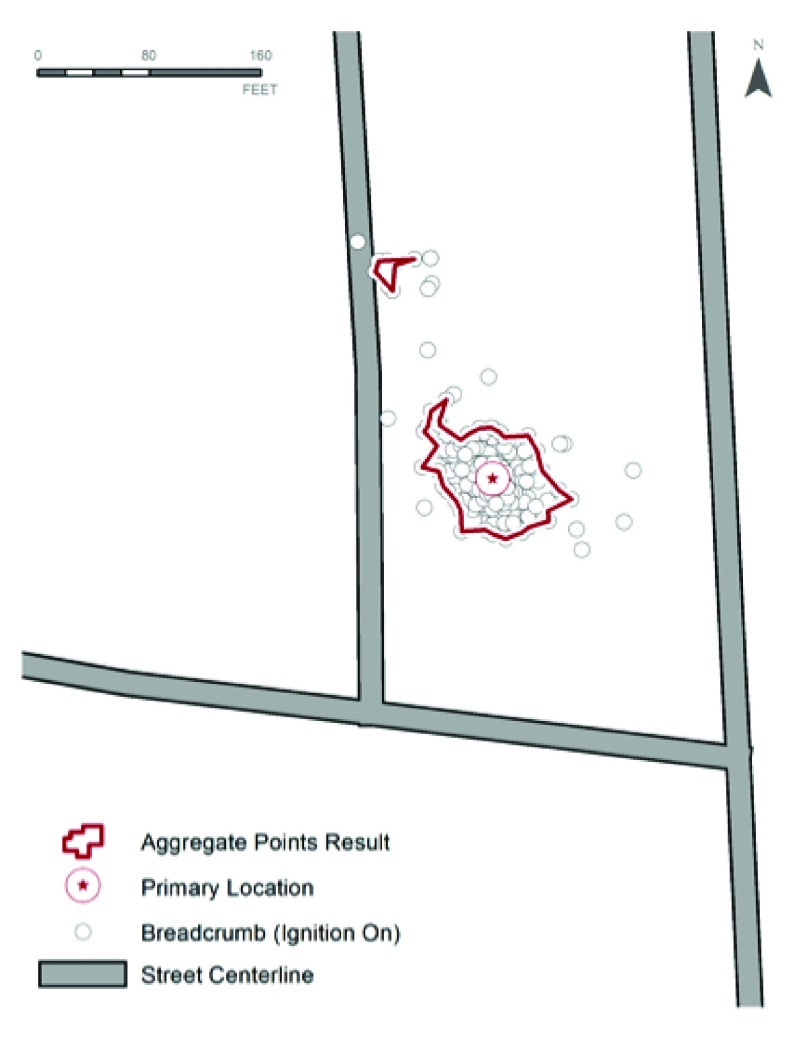
A primary location polygon created using the aggregate points geoprocessing operation with a primary location point placed at its centroid.

**Primary Locations.** The participants’ most commonly-visited locations (Primary Locations) were identified in order to perform spatial analysis on aspects of the drivers’ behavior relative to familiar areas. The participant’s home and/or workplace were assumed to be the most frequent origin or destination of the majority of the trips recorded by the GPDAS. It was crucial that these locations be identified in a dynamic and automated way to achieve scalability of the data processing workflow. A visual examination of the data for a small sample of participants showed that an often-visited location could appear as a dense cluster of breadcrumbs. It was assumed that the densest cluster, or the cluster with the most ignition on breadcrumbs, would be the Primary Location. Clusters of ignition on breadcrumbs were identified using the Aggregate Points geoprocessing operation. The Aggregate Distance parameter was set to 20 feet after visually locating and measuring ignition on breadcrumb clusters on a small sample of participants. The output of the Aggregate Points operation was polygon feature class with features encompassing clusters of three or more points within the Aggregate Distance parameter value. The breadcrumbs located within each polygon were counted and compared to the total number of ignition on breadcrumbs for the participant to determine if the polygon represented a Primary Location. The Feature To Polygon geoprocessing operation was used to produce a point feature at the centroid of each Primary Location polygon, thus providing a single point that was used as the Primary Location in further analyses ( Figure 3).

**Unique destinations.** Unique destinations are defined as separate locations visited by participants during a given timeframe. The Buffer geoprocessing operation was used to create circular polygons with radii of 100, 250 and 500 feet around each breadcrumb indicating an ignition on event. The varying buffer operations were performed to establish a threshold at which two or more distinct breadcrumbs occurring within the same radius during the same time period would be combined as the same destination. For example, a participant who visited a shopping center twice in the same month may park at opposite ends of the large parking area for each separate visit. However, this shopping center should be counted as a single destination for the target time period.

**Figure 4.**
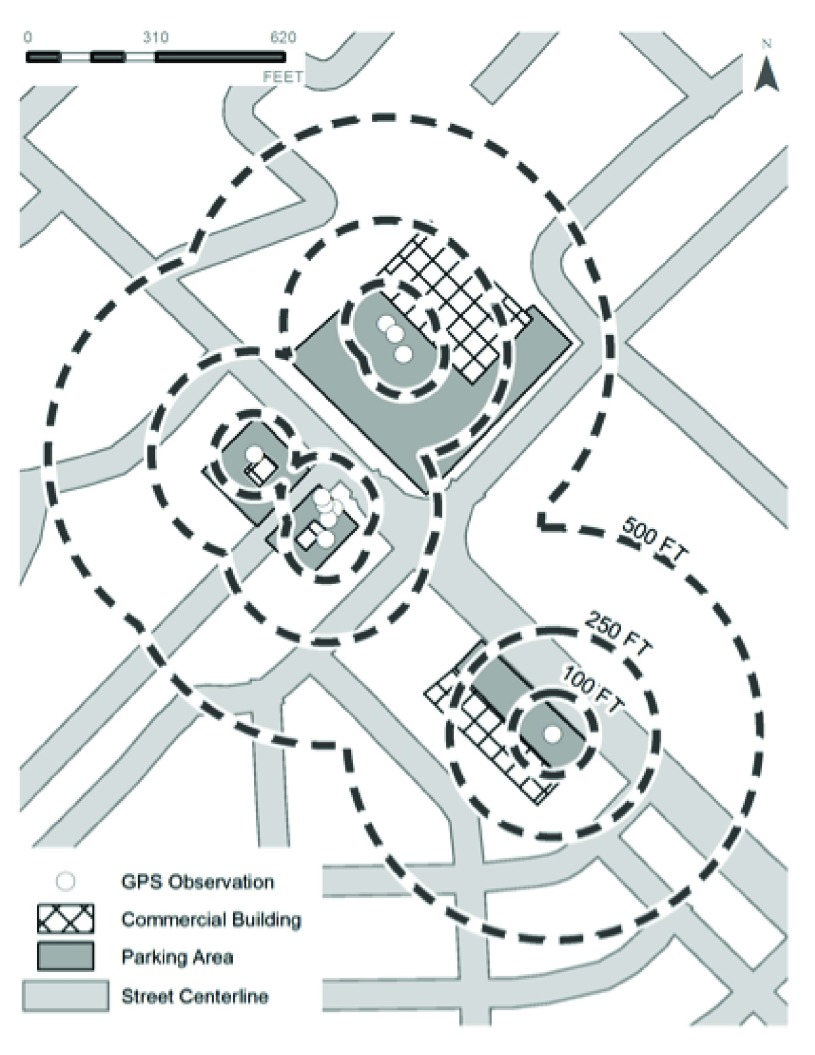
Unique destination sample with multiple buffers.

The Dissolve geoprocessing operation was used to merge the circular polygons so breadcrumbs within the three distance thresholds would be counted as a single destination. Figure 4 shows a sample of ignition on breadcrumbs in a selected area during a single month. The groups of breadcrumbs within close proximity to each of the commercial buildings occur on different days within the same month. The 500 foot buffer polygon encompassed all four separate commercial destinations and would be counted as a single destination for that month. The 250 foot buffer would combine the northernmost commercial area with the two areas to the southwest, creating a single destination from three distinct destinations. The 100 foot buffer separated the three separate destinations into two destinations, combining only the two smallest commercial areas into a single destination ( Figure 4).

## Results

**Comprehensive driver profiles.** A breadcrumb is one data point in time (at 30-seconds interval) that contains location, time, date and speed of a vehicle. A single trip could have hundreds of breadcrumbs that are aggregated and over time can provide specific information about driving patterns and behaviors. The steps discussed in the methodology section resulted in the creation of a driving profile for each driver that could be examined over the course of a study. Driver profiles included spatial, temporal and behavioral components. Spatial components included Primary Location(s), Driving Areas, Mean Centers and Unique Destinations. Temporal components included number of trips taken during different times of day. Behavioral components included number of hard braking, speeding and sudden acceleration events. Across the five months, the mean and standard deviation for the total number of trips was 552.7 (209.5) and for average miles per trip was 6.8 (3.2).

**Figure 5.**
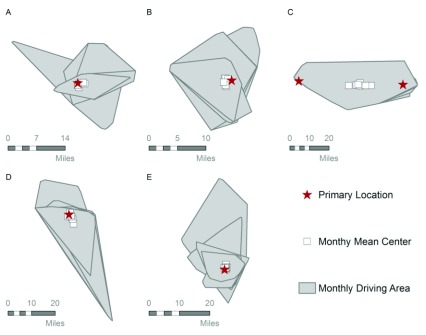
Spatial profiles.

**Primary Locations.** A driver’s Primary Location was designated as the location that encompassed at least 10 percent of the driver’s Ignition On breadcrumbs. Since participants are over the age of 65, in most cases, participants had a single Primary Location, assumed to be their home/residence, though some participant results showed two Primary Locations. In most cases, the count for the cluster polygon with the highest count of breadcrumbs was significantly higher than the counts for the other two polygons. The exception is Participant C, where two cluster polygons have breadcrumb counts over 10 percent of the total breadcrumbs ( Figure 5). Participant C has two Primary Locations based on the percentage of driver’s ignition on events. Primary Locations were compared against the known addresses from the participants and confirmed to be 100 percent accurate, including participant C who is known to have two homes.

**Driving Area and Mean Center.** The Driving Area polygons resulting from the methodology varied based on the extent of the breadcrumbs for each driver over time. Analysis showed that a participant's driving areas could often have large portions of overlap from week to week or month to month. Mean Centers were expected to be clustered around the participant’s Primary Location. However, this was not the case when participants had more than one Primary Location. See Participant C in Figure 5. Participant C had two designated Primary Locations and as a result, the Mean Centers for this participant tend to be located between the two Primary Locations. The combined Driving Area polygons, Mean Centers and Primary Locations make up the spatial profile for study participants. Spatial profiles for a sample of participants are visualized in Figure 5. Each grey polygon represents the driving area for a single month for each participant. Monthly Mean Centers are represented with white boxes and red stars indicate the Primary Location for each participant.

**Figure 6.**
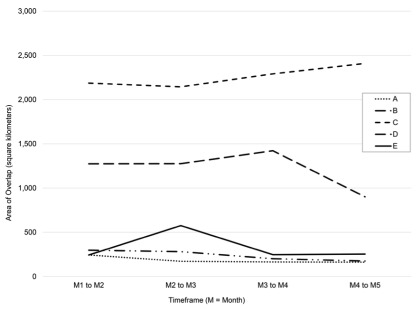
Area of overlap between Driving Areas from month to month.

Driving Areas can vary greatly month to month for some participants, while other participants tend to have little monthly variation in their driving area. The monthly Driving Area polygons for participants C and D show large portions of overlap, while participants A, B and E show large portions of Driving Area unique to a single month timeframe. Common Driving Area can be quantified by calculating the overlapping area from month to month and overall overlapping area for the five-month study period. The month to month variation in overlapping Driving Area is shown in Figure 6 and reinforces the large amount of overlap from month to month for participants C (first line from top) and D (second line from top).

**Figure 7.**
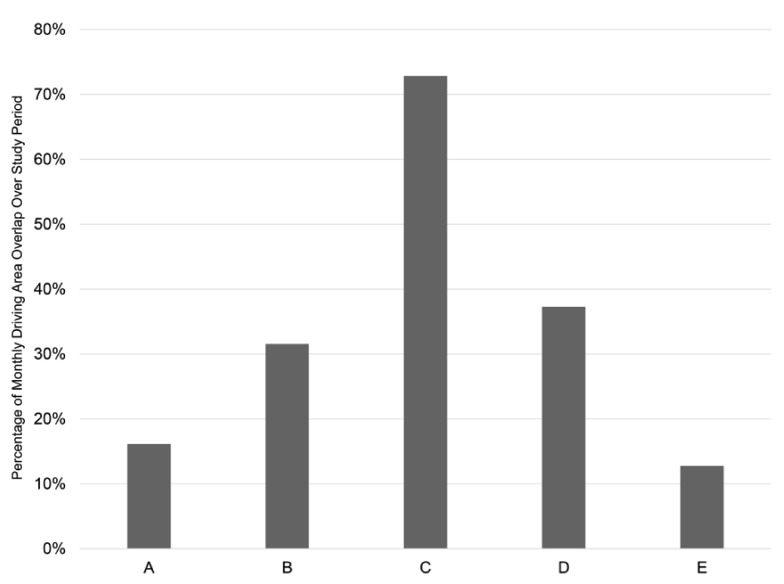
Ratio of overlapping driving area to total driving area over the 5 month period.

The ratio of overlapping Driving Area over total Driving Area examines the relationship between commonly driven routes and total driving space. In Figure 7, participant C shows little variation in monthly driving area during the study timeframe with over 70% of the total Driving Area being common to all months. Participant E shows the least amount of overlapping area with less than 15% of the total Driving Area being common to all months.

**Figure 8.**
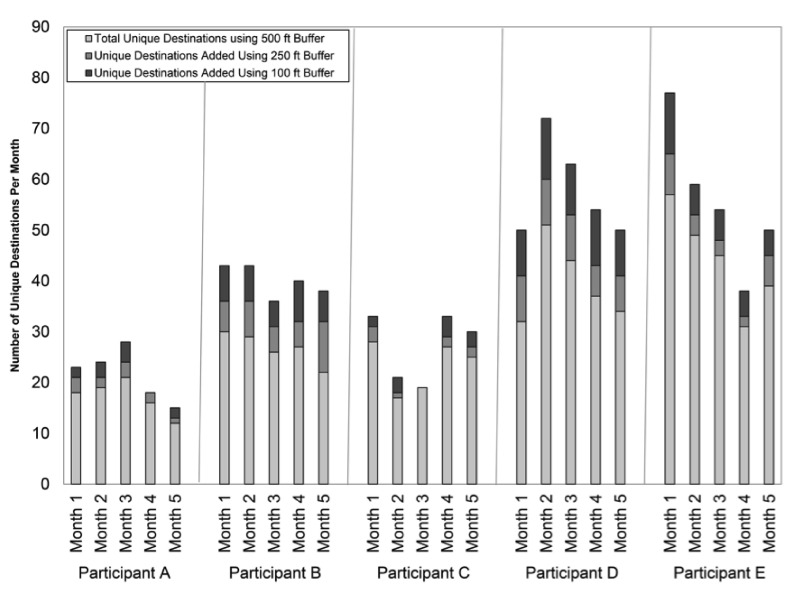
Counts of unique destinations for 5 participants.

**Unique Destination.** The results of performing the Unique Destinations methodology showed varying results by driver. While some drivers showed similar counts of Unique Destinations each month, other drivers showed counts of Unique Destinations that varied greatly from month to month ( Figure 8). In most months for many drivers, the counts of Unique Destinations derived by using the 100, 250 and 500 feet buffers varied by buffer size. However, if a driver’s destinations were particularly spread out, the buffer size was less consequential. Overall, the results show that the 100 feet buffer should be used to obtain the most accurate count of unique destinations for the participants within each time period.

**Figure 9.**
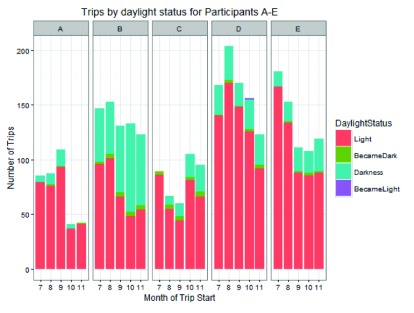
Number of trips driven during day and night.

**Trips driven in daylight vs. night-time.** The results of the number of trips driven during the day vs. night showed variation across individuals and intra-individual change across different months. Figure 9 displays the number of trips driven during day and night for five participants from July (7) to November (11). Night driving is associated with a three times greater risk of traffic death and increased fatigue and perceived danger ^13, 14^. The majority of trips driven by 4/5 participants were driven during the day. For participants B and D, the number of trips generally declined from month 1 to month 5 without a significant change in their number of trips driven at night. However, Participant C had a higher total number of trips for months 4–5 and increased night driving compared to months 5–7. Participant A reduced their night driving and total number of trips taken from month 3 to 5 while participant B showed little change in night driving behavior. Given that the time window of our study represents months when the hours of daylight available are steadily decreasing, the decrease in total number of trips combined with the lack of a corresponding increase in trips taken at night may suggest that the driver A in our study made deliberate adjustments to avoid night-time driving. Finally, more trips were started during dusk, compared to dawn.

**Figure 10.**
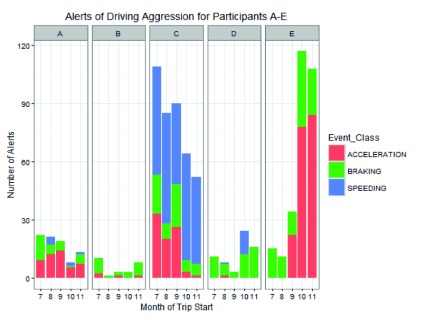
Total number of alerts across the 5 months.

**Adverse driving behavior.** Three alerts (speeding, hard braking, and hard acceleration) were identified by the GPDAS that reflect adverse driving behavior independent of the environmental driving context. Speeding was defined as driving more than ≥6 miles per hour above posted speed limit in an area. Duration and maximum speed were gathered and available from the device, however, only the counts of alert were reported here. Hard braking and hard acceleration were defined as a change (decrease/increase) in speed of 8 to 10 miles per hour in one second, respectively. Counts were also reported for hard braking and hard acceleration. All definitions were adopted from the insurance industry by the device vendor. The GPDAS does not capture data on traffic flow or congestion, weather patterns, inclement conditions, or other factors ( *e.g.* altered mental state) that may impact the driver behavior. Figure 10 presents data on hard braking, sudden acceleration and speeding for five participants across the five months. Similar to the spatial and temporal analyses, there was a wide variation among the participants. The difference between the least and most aggressive drivers shown here is dramatic: Participant B recorded 25 total alerts while Participant C recorded 400. Participants B and D had no speeding alerts, while participant C recorded all three types of aggressive driving patterns in all 5 months. Participant D recorded three times as many braking events as speeding and sudden acceleration events combined. In months 10 and 11, Participants D and E show a marked increase in aggressive habits, while Participants A and C seem to decline in aggression. The inter-individual variation in driving alerts over the five months may be a reflection of driver preference or style, the driving environment or the interaction between both. While the data presented in Figure 10 is a total count of alerts, it is possible to examine the frequency of trips containing one or more alerts. It is unlikely that the high number of alerts among some participants ( *e.g*. C, E) may be solely attributed to the driving environment.

In summary, the COTS GPDAS device was able to capture objective driving behavior. Data obtained provided a foundation for creating a Naturalistic Driving Profile that included spatial, temporal and behavioral components. This methodology allows us to track a number of variables describing the driving behaviors and patterns of participants over time. We were able to confirm the accuracy of the methodology of identifying the Primary Locations by comparing the results to the actual addresses reported by the participants. Based on the locations of the breadcrumbs, we were able to successfully identify frequently visited locations and general travel patterns. Based on the reported time from the breadcrumbs, we could assess number of trips driven in daylight vs. night-time. Data capturing special events allowed us to compute the number of adverse driving alerts over the 5-month period.

## Discussion

This pilot study presented the feasibility of adapting a COTS GPS device to examine daily driving behavior and associated changes in a cohort of cognitively-normal older adults. The ability to understand changes in driving behavior in the actual environments people drive has been unavailable until recently.

The GPDAS provided continuous driving data that was used to develop a unique Naturalistic Driving Profile combining spatial, temporal and behavioral aspects of driving. Specifically, we were able to obtain date, time, location and a set of metrics that balanced the ability to measure consistency and change in driving behavior, without over-collecting data or over-burdening research participants. The complexities and obstacles of working with large datasets have been well documented ^15^. The key methodological challenges in this research included: 1) synchronizing data collection from the GPDAS and the vendor servers, 2) efficiently processing and error checking the ‘big data’ on a daily basis 3) developing data cleaning procedures for common errors ( *e.g.* device removal or signal loss) and uncommon errors ( *e.g.* device failure) and 4) synthesizing the data for management and analyses in R and ArcGIS.

 This naturalistic driving methodology provides several advantages to understanding driving behavior over conventional methodologies. The GPDAS can be used to simultaneously monitor real-time driving behavior in a large cohort across the continental United States. The GPDAS’ great strength comes in being able to observe individuals and compare intra-individual change over a long period of time. Additionally, the ease of installation (less than 1 minute), no vehicle modification, minimal effort from participants and seamless data acquisition and transmission strengthens its utility.

Given this was a COTS GPS device, there are some limitations with using this device and methodology. Recent naturalistic driving studies have implemented and used more sophisticated systems including cameras, installed hard disk drives and a combination of sensors hardwired to a vehicle’s engine and wireless sensors ^16– 18^. While we sampled data every 30 seconds, other studies have sampled data every 1 to 5 seconds to increase the fidelity and robustness of their results ^19^. The length of this pilot was shorter when compared to other studies (e.g. CanDrive) that have published driving data from several years of data collection ^20^. Some studies have used radio frequency identification tags to identify the driver and anonymize home locations of their participants ^21^. Driver identification was limited to participant self-report. The vendor now offers a Bluetooth Low Energy (BLE) beacon the size and weight of a credit card that may be placed in a wallet or purse. The BLE beacon automatically pairs with the GPDAS when the participant is in the driver seat to identify the driver. This simple solution is automatic, requires no participant effort, conveniently syncs with the devices data stream and is downloaded with the device’s data. We were not able to detect under-speeding as robustly as we had hoped, due to a variety of confounding factors such as traffic, construction speed limit changes, and the granularity of the breadcrumbs. The unknown product life of GPDAS devices poses a particular challenge for longitudinal naturalistic driving studies. We detected several potential warning signs for device failure, and were able to take proactive steps to order replacement devices when failures were detected. However, such replacement is not simple for some study participants since it requires travel to our facility for a new device and may reduce their willingness to remain in the study. Finally, it is important to consider the goals, outcomes and amount of participant burden when selecting a methodology for longitudinal studies assessing driving performance and behavior.

**Ethical approval and consent to participate:** All participants were recruited and tested at Washington University School of Medicine. Written informed consent to use and publish clinical details was obtained from all participants. All aspects of the study were approved by the Washington University Institutional Review Board.

Creating a driving profile for older adults using GPS devices and naturalistic driving methodology Dataset legend: id: Participant ID; numofdays: Number of days driven; numtripsover5mo: Number of trips driven over 5 months; trips_at_night5mo: Number of trips driven at night over 5 months; trips_w_HB5mo: Number of trips driven with hard braking events over 5 months; trips_w_SA5mo: Number of trips driven sudden acceleration events over 5 months; trips_speeding5mo: Number of trips driven with speeding events over 5 months; hours_speeding5mo: Number of hours speeding over 5 months; tot_dist_driven5mo: Total distance (miles) driven over 5 months; tot_drv_hrs5mo: Total number of hours driven over 5 months; avg_trip_miles5mo: Average trip miles over 5 months; avg_trip_mins5mo: Average trips minutes over 5 months; trips_at_night: Number of trips at night over 5 months. Click here for additional data file. Copyright: © 2016 Babulal GM et al. 2016 Data associated with the article are available under the terms of the Creative Commons Zero "No rights reserved" data waiver (CC0 1.0 Public domain dedication).

### Data availability

F1000Research: Dataset 1. Creating a driving profile for older adults using GPS devices and naturalistic driving methodology, 10.5256/f1000research.9608.d135843 ^22^
