# Supplementary material for: Creating a driving profile for older adults using GPS devices and naturalistic driving methodology
Source: F1000Res. 2016 Dec 7;5:2376. Originally published 2016 Sep 26. [Version 2] doi: 10.12688/f1000research.9608.2 (PMC5133689; doi:10.12688/f1000research.9608.2)
Supplement: Peer review history

# Peer review: Referee response for version 1

**DOI:** 10.5256/f1000research.10351.r17017

**Published:** 2016-11-29

**Authors:** Monique M. Williams

**Affiliations:** 1 VITAS Healthcare, St. Louis, MO, USA

The authors describe their adaptation of a commercial off the shelf (COTS) in-vehicle device that captures GPS date. These data are analyzed using GIS techniques.

 Data were collected from a total of five participants who were members of a longitudinal cohort study. The five participants were aged 65 years and older and were cognitively normal. They possessed a valid driver’s license and drove at least once a week. In addition, all participants had provided Alzheimer’s disease biomarker data (brain imaging or cerebral spinal fluid studies) in the past two years.

 The authors used COTS Azuga G2 Tracking Device for data collection. The data included longitude, latitude, and vehicle speed. Data were collected every thirty seconds from the time that the ignition was turned on. In addition, aggressive driving behaviors such as speeding, hard braking, and sudden acceleration were logged. A driving profile was developed for each participant. The profiles included spatial, temporal, and behavioral components.

 This pilot study demonstrates that the COTS device provides accurate data regarding daily driving behavior in older adults. The driving profiles for participants can be compared month-to-month or year-to-year and thus, provide the opportunity for observing changes in driving behavior.

 With the aging of the population, the pilot study provides data that can be applied to the development of further studies to provide additional characterization of driving profiles of older adults.

I have read this submission. I believe that I have an appropriate level of expertise to confirm that it is of an acceptable scientific standard.

# Peer review: Referee response for version 1

**DOI:** 10.5256/f1000research.10351.r17216

**Published:** 2016-10-26

**Authors:** Xueqin Qian

**Affiliations:** 1 University of Minnesota, Department of Educational Psychology, Minneapolis, MN, USA

The authors adapted an in-vehicle device to measure driving behavior in individuals with Alzheimer’s disease. Data from five participants were collected using the COTS Azuga G2 Tracking Device. Results from this study suggest that this technology was able to accurately identify locations and travel patterns of the participants.

 Given the increasing number of individuals diagnosed with Alzheimer disease and the important role that driving plays in a person’s life, this study has the potential to provide a measurement tool that can be used reliably to study driving involving seniors with Alzheimer disease. I have two recommendations for the authors to consider: 

1. For the introduction, can authors provide a little background information on the driving behavior of senior citizens with Alzheimer? It may be helpful to orient readers who are unfamiliar with this topic.
2. As for results section, do you have data on under what situations, the alert behaviors happen the most frequently? If so, would it be possible to add it to the result section?

I have read this submission. I believe that I have an appropriate level of expertise to confirm that it is of an acceptable scientific standard.

# Response

**Published:** 2016-11-28

**Authors:** Ganesh Babulal

**Affiliations:** Washington University School of Medicine, USA

Dr. Qian: Thank you for your prompt review and comments on this manuscript.

 On your first point—since this is a methodological article, we limited the amount of information on driving performance and Alzheimer’ disease in the background section. However, we have added a sentence in the introduction with a recent reference (2016) of a systematic review that summarizes the evidence on driving in early stage AD.

 On your second point—we have the location (latitude and longitude), time and date of where/when the alert for adverse driving behaviors occurred. We are working to analyze these breadcrumbs, but given the high volume of data for each participant we do not have results available at this time that can be added into the result section of the current manuscript.

# Peer review: Referee response for version 1

**DOI:** 10.5256/f1000research.10351.r16603

**Published:** 2016-10-21

**Authors:** Frank-Dietrich Knoefel

**Affiliations:** 1 Bruyère Research Institute, Ottawa, ON, Canada

With the current lack of an accepted standardized approach to assessing changing driving risk associated with aging, and with emerging technology, naturalistic driving is an important area of research. The authors of this paper have a strong track record in publishing in the field of aging, cognition and driving.

 This paper provides the results of a pilot study of 5 participants using an off-the-shelf global positioning data acquisition system connected to the OBDII port of the older driver’s car. It describes how 5 months of data, collected by the company selling the device, was cleaned and processed to identify such features as spatial components (primary location, unique destinations and driving areas), and temporal components (time of day by season). They also used data obtained from the company on adverse driving behavior: speeding, hard breaking and hard acceleration. Using these features they were able to show differences between the drivers. It may be implied that changes in these parameters over time could be used in the future to help assess changing driving risk or help determine interventions to keep older adults driving longer, safely.

 Major issues:

- While this paper is well-written and describes in some detail the work this group has done, it is not clear what the new contribution of this work is. While the Background suggests that the objective was to “describe methodological challenges associated with adapting a COTS in-vehicle device that captures and synthesizes GPS data for processing and analysis using GIS techniques” the abstract does not refer to this.
- Importantly, the design of this sensor system and analysis of data were not compared to other systems recently described in the literature, for instance by Marshall *et al.* (CanDrive project), Eby *et al.*(2012), and the SHRP2 study by Skog & Handel (2014). Specifically, work on destination identification has been published by Wallace *et al.* (2013). Our group has further addressed the issue of how to anonymize locations to protect individual driver identity (especially home address), a requirement of most research ethics committees (Wallace *et al.* 2015). Data cleaning has been discussed in papers by Porter and Wallace. The sensor information (breadcrumbs) logged every 30 seconds should be compared to other groups, for instance we sampled data every 1 to 5 seconds. The length of the pilot data is short compared to our published work using 1 year of driving data, from 7 years of available CanDrive data set. Finally, Wallace *et al.* (2014) have published work on driver identification that is not referenced
- Similarly, to be able to compare to other literature we need the definitions used for the alerts, e.g. how much over the speed limit was considered “speeding” (absolute or percent), and how this was determined (via GIS data?). Similarly we do not have definitions for hard accelerating and hard braking.

Minor issues:

- Background

Page 3: “recent studies are limited to capturing data… from weeks to 2 months.” CanDrive has collected driving data for 7 years from several hundred drivers – downloaded every 4 to 6 months.

- Methods

Unique destinations paragraph: for clarity: suggest “two or more distinct breadcrumbs occurring within the same radius during the same time period would be combined….”

 I would move the sections describing the results of breadcrumbs (Fig 2) and the section how changing the buffer from 100 to 500 feet affects the grouping of breadcrumbs (Fig 3 and 4) into the Results section.

- Results:

The Abstract refers to 5 participants as a subset of the study participants, but we don’t have a description of these drivers (age, gender, cognitive scores).

 The first paragraph states: “A single trip could have hundreds of breadcrumbs that are aggregated and over time can provide specific information about driving patterns and behaviors.” Why were we not given the mean and range of breadcrumbs for trips, or time and distance of trips?

 Figure 6 – legend: the lines for participants B and D look similar.

 Page 7: Figure 6 is referred to twice – the second time should read Figure 7 (percentages).

I have read this submission. I believe that I have an appropriate level of expertise to confirm that it is of an acceptable scientific standard, however I have significant reservations, as outlined above.

## References

[1] EbyDWSilversteinNMMolnarLJLeBlancDAdlerG: Driving behaviors in early stage dementia: a study using in-vehicle technology. *Accid Anal Prev*.2012;49: 10.1016/j.aap.2011.11.021 330-7 10.1016/j.aap.2011.11.021 23036413

[2] MarshallSCMan-Son-HingMBédardMCharltonJGagnonSGélinasIKoppelSKorner-BitenskyNLangfordJMazerBMyersANaglieGPolgarJPorterMMRapoportMTuokkoHVrkljanBWoolnoughA: Protocol for Candrive II/Ozcandrive, a multicentre prospective older driver cohort study. *Accid Anal Prev*.2013;61: 10.1016/j.aap.2013.02.009 245-52 10.1016/j.aap.2013.02.009 23541299

[3] SkogI.HändelP.: Indirect instantaneous car-fuel consumption measurements. *IEEE Transactions on Instrumentation and Measurement*.2014;63(12) :3190-3198 Reference source

[4] WallaceB.GoubranR.KnoefelF.: Cognitive change measurement through driving navigation ability sensing and analysis. *In Medical Measurements and Applications Proceedings (MeMeA), 2013 IEEE International Symposium on*.2013, May; 10.1109/MeMeA.2013.6549728 164-169 10.1109/MeMeA.2013.6549728

[5] WallaceB.GoubranR.KnoefelF.MarshallSPorterM.: Measuring variation in driving habits between drivers. *In Medical Measurements and Applications (MeMeA), 2014 IEEE International Symposium on*.2014, June; 10.1109/MeMeA.2014.6860025 1-6 10.1109/MeMeA.2014.6860025

[6] WallaceB.GoubranR.KnoefelF.MarshallS.PorterS.HarlowM.PauliA.: Automation of the Validation, Anonymization, and Augmentation of Big Data from a Multi-year Driving Study. *2015 IEEE International Congress on Big Data (BigData Congress)*.2015, June; 10.1109/BigDataCongress.2015.93 608-614 10.1109/BigDataCongress.2015.93

# Response

**Published:** 2016-11-28

**Authors:** Ganesh Babulal

**Affiliations:** Washington University School of Medicine, USA

Dr. Knoefel: We thank you for your thorough review and the additional references. We have made the following edits to the manuscript in consideration of your comments:

1. We have added in text into the abstract to clarify our objective for this paper.
2. We added information comparing the GPDAS device and data collection process to prior research studies that used naturalistic driving methodologies. The references have also been added as appropriate to the text.
3. We have added definitions for the alerts of speeding, hard braking and hard acceleration in the results section under the subsection, adverse driving behavior.
4. The sentence in the background section was modified to point out that some, not all studies have a short collection interval. We also referenced the CanDrive and its extensive data collection interval in the discussion section.
5. The clarification was made in the methods section to specific radius. We chose to retain the structure of the methods section to reflect a similar layout in the results section.
6. Demographics on the five participants were not included in this methodology article since the focus was on the GPDAS device, data collection and processing. Results examining differences among participants in driving behavior will be published later after more driving data is collected.
7. The mean and standard deviation for total number of trips and average miles per trip have been added for the group of participants.
8. We have also clarified the graph lines for the participants B and D in the text and corrected the reference to Figure 7 in the text.
